# Natural or Supernatural?—“Spirits” or Telepathy?

**Published:** 1910-10

**Authors:** F. E. Daniel

**Affiliations:** Austin, Texas


### NATURAL OR SUPERNATURAL?—"SPIRITS" OR TELEPATHY?

BY F. E. DANIEL, M. D., AUSTIN, TEXAS.

It has ever been the tendency of the human mind to attribute to supernatural causes phenomena which can not be explained or accounted for by natural laws. It has not been a great many years since all electrical phenomena were so regarded, and at one period in the world's history even thunder and lightning were said to be an expression of the "wrath of Jove." To this day, the ignorant assign to some extra natural agency certain phenomena which the more enlightened understand perfectly well. And this is quite natural. But in this age of enlightenment and progress in every line of human thought and achievement—unless I except theology—it is to me most surprising that educated men,—men of strong mentality, vigorous intellect and reasoning powers cultivated and stimulated by study, reflection and daily exercise and practice,—should still hold on to the worn-out and exploded doctrine of "spiritism" or "vitalism." These dogmas of the church dominated the intellectual world for ages and kept the human mind in the bonds of superstition. They held that man's life, or "spirit" was, "from himself a thing apart"; a something—nothing,—external to man, which entered his body at birth, dominated it, controlled it, shaped his acts, and at death left the body and maintained a separate existence for all time and eternity. To hear able, and otherwise sensible men speak seriously of "disembodied spirits," and of "communications from the dead to the living" strikes me as being absurd. For my part, I utterly repudiate all such agency as "spirits." It is superstition, pure and simple. The "dead" can not "communicate with the living." There is no such thing as "spirit," any more than there are "angels" with material wings; both are purely mythical. It is transcendental. Man can not know anything except through his senses. Hence, all knowledge is empirical. He can conceive of nothing that he can not symbolize in his mind, hence the attempt to symbolize God results in the anthropomorphic idea of God. True, man can have a reasonable faith in the existence of things which he can not know,—which can not be rendered tangible to any of his senses. He can arrive at conclusions, often correct, by reasoning from the known to the unknown. By inductive reasoning he "knows" that atoms exist, that ether exists. No man ever saw an atom; but there is reason for the belief or faith that atoms do exist. Matter (mass) may be divided and subdivided until the vanishing point is reached.

In this way, reasoning from observed effects on the neighboring planets and without ever looking into the sky, Leverrier reached the conclusion that there was a large body outside of and beyond the orbit of Uranus. He sent his calculations to the observer at Berlin, and told him to turn his telescope to a certain point in the heavens at a certain hour, and he would find a large planet, which, he was sure, must be there. Behold, the discovery of Neptune.

There is no reason for the belief in "spirits," and no proof or evidence can be adduced in support of the belief. It is contrary to reason and common sense. It is a falling back upon the supernatural to account for the phenomena which, in the present state of the human mind and knowledge are inexplicable, but which, in time, will be understood and accounted for by natural laws. The belief in "spiritual beings," "disembodied spirits,"—"immaterial substance," it is called (a contradiction of terms), is not hard to account for on the principle of heredity and (misdirected) education. The idea of a "spiritual being" without form or shape —(concepts of the human mind)—and having no corporeal existence, but yet is credited with the physiological functions of a material organism—finds the culmination of absurdity in that dogma which ascribes to a "spirit" or "ghost" (called "holy") the powers of reproduction of the human species—purely a human physiological act. Men laugh at the amours of the mythical Jupiter of the Greeks, whose chief pastime seems to have been the begetting of children my men's wives, yet they accept in all seriousness the statement that the Jupiter of the Jews, "Jove," "Jehovah," "Yehveh," the myth upon which the Christian religion is founded, "overshadowed" a "virgin" and begot a son, who, like the progeny of Jupiter, at death, "ascended into heaven" and became a god. This is superstition in its rankest form, a "miracle," and yet learned men believe it. Parthenogenesis ("the creation of a new individual of a species from a female cell without the intervention of a male element") is an established fact in biology, but has never been known to occur in man or any of the higher animals, but it does occur in certain plants, and in certain of the lower forms of animal life, notably in bees. (Alternate generation. Heterogamy.)

The late Professor Norman, of the University of Texas, at one time thought that he had produced young sea urchins by chemical means from the unfertilized ova of the female; and Professor Loeb, of the University of Chicago, is now engaged, and with hope of success, in endeavoring to produce, synthetically, the "male element," whereby he expects to fertilize the ova of the sea urchin, and thus produce a living animal.

Surely those learned gentlemen who hold to the faith ("faith, the sleep of reason") in a spiritual existence, and talk and write learnedly of "disembodied spirits," attributing to them the physiological functions of the living organism, have not made themselves acquainted with the wonderful advances and discoveries in physiology, chemistry, embryology, anatomy, and in general physics, that have been made and become public property in the last half of the nineteenth century. I will speak of this later.

I take the mechanical view of life, that held by most physicists and biologists, and taught by the great expounders of the monistic philosophy, and of what has come to be known as the Science of Energy or Energetics.

I assume that those who read this are sufficiently familiar with the Law of Conservation of Energy and Correlation of Forces, promulgated (1845) by Robert Mayer, a young physician at Wurttemberg, and elaborated and forever firmly fixed in the mind of all scientists by Helmholz in his celebrated essay on the subject; and with the Law of the Indestructibility of Matter (Lavoisier, 1789), "nothing is created, nothing is lost, all is change of forms," —and I need not do more than allude to them, further than to say that the Science of Energetics applies to all phenomena, seeks to explain all phenomena of the universe, including those of human life. It embraces and unifies all other sciences, and has brought or will bring them under its dominion. "According to most physicists, the phenomena of the universe call into play, two, and only two, elementary and fundamental things; towit, matter and energy. All that we see consists of changes in the one or the other of these two forms. This, one might say, is the postulate of experimental science." (A Dastre, *Revue des Deux Mondes,* 1898.)

I need not say that all energy is derived from the sun; nor more than allude to the reciprocal functions of animal life and plant life. Plants store the sun's energy, and we find it, after millions of years, still stored in the coal we dig from the bowels of the earth, where it has remained all these centuries in the latent or potential state. Plants split up the carbon dioxide of the air, freeing the oxygen,—man's vital necessity and that of all breathing animals, and store the carbon in wood, starch and sugar. It is the action of oxygen on the carbon, whether in coal or wood, or in our own aliment (essentially carbon and nitrogen compounds), which liberates this energy, converting it from the latent, or potential state, into active (kinetic) energy. This, in the animal economy, is subdivided or specialized, and expressed in the various well known forms of "radiant" energy, "thermal" energy, "kinetic" or moving energy, "chemical" energy, and "electric" energy (including magnetic). These forms are all correlated, and are intro-convertible, transformable, the one into the other, except heat energy. That is not transformable directly, into any other form of energy. Take the locomotive for an illustration. The combustion of the coal put into the furnace (a chemical process, oxidation) liberates the potential energy stored in the coal by the sun. It is expressed in those "modes of motion" (molecular) known as heat and light. The heat changes the molecular arrangement of the water in the boiler, converting it into an expansive vapor, which, being confined, seeks to escape, and thus drives the piston which moves the engine and train (mechanical energy, kinetic energy). The steam also propels the dynamo, whereby a part of this liberated energy is "specialized," and transformed into electric energy, which is expressed in the headlight that blazes on the locomotive's front, and in the incandescent light, whereby the coaches are lighted.

Man's energy (his life force) and that of all animals is derived from the food put into his stomach, and from the reserves of nutrition already stored in his tissues. All food that has nutritive value is essentially carbon and nitrogen in endless combination. The liberation and conversion of this latent (potential) energy is effected by chemical means, oxidation, combustion, and it is expressed in all the phenomena associated with the life of the animal; heat, muscular motion (kinetic energy, mechanical or "work" energy); secretion, digestion, assimilation, metabolism (destructive and constructive*) and consciousness, the fundamental state upon which rests the mind, with its multifarious

---

*The power that organized bodies possess of continually using up and renewing the matter composing the body, Cuvier's "vital vortex," the endless circulation of matter and energy from the outside world through the human system by ingestion, secretion and excretion.

phases or functions of thought, volition, memory, ideation, all the higher intellectual faculties. How consciousness arises, no one knows, any more than he knows how "life" began—what gave to "matter" (protoplasm, the primitive cell), sensation, motion, and I am sometimes disposed to think, intelligence, consciousness. But consciouness is doubtless a product of the food taken into the stomach. It is a part of the life energy liberated by the chemical reaction between carbon compounds of the aliment (and the tissue reserves) and the oxygen taken in through the lungs, and is specialized or told off for special work, by the wonderful electro-chemico dynamic apparatus we call the "nervous system," properly, the net-work of special cells (neurons) that ramify in and connect every part of the body. It is electrical. The mind, in all its phases, is the function or product of the brain. It is a force, a specialized force, and is electrical. We see, hear, feel, think and act by electricity. The touch, the thrilling kiss, is but an electrical discharge. This force, the mind, can be directed by the will. It is a power capable of overcoming gravitation and lifting ponderable matter, to some extent. We have the authority of Elliott Coues for saying that he has seen three legs of a heavy dining table leave the floor, when he and his wife were alone in the room, and without the application of any other force than that of the mind. He calls it "levitation." It can be directed towards a person, say, across the hall, or church, or theater, and that person be made to feel it. It will wake a sleeping person to look at him—the force being expressed through the eyes. We all know how an eloquent speaker can sway men's thoughts, and move them to overt acts,—even to commit violence.

The mind, then (and, when I say mind, I mean all that is understood by the term), including what is called the "soul," whatever that may be thought to be, is electrical energy, generated by chemical action, and is purely mechanical; it is a mode of motion, generated by a living organ, the brain, and as surely ceases when the combustion which has produced it ceases, when the brain dies, as does the flame of a lamp when the oil or wick gives out. I can as nearly understand and believe that the light of the incandescent lamp by which I pen this, exists and persists, for all time, as this special and individual, separate light, as that a life force, generated in precisely the same way (combustion, oxidation of carbon) and specialized into the electrical form of energy, can continue after the death of the organ, and consequent cessation of the processes whereby it was produced. It is now known that all animal life is of a kind, differing only in degree of development; and if a man has a "spirit' (*spirare,* to breathe), or a "soul" (*psucho*, breath, to breathe), every breathing animal, at least, has a spirit or soul. In fact, the line can not be drawn anywhere from the amoba or the moneron, to man.

I conceive of life, then, in all its phenomena, including the psychic, as chemical in its origin, chemical in the union of the male and female cell in utero—an expression of chemical affinity in exact fulfillment of an immutable law, and that the building up of the resulting embryo—and fetus—and child—and finally the man, is a purely mechanical process; the result of combustion, the conversion of potential into active energy which is expressed in all the various modes of molecular motion, kinetic energy, radiant energy, thermic energy, electrical (including magnetic) energy; and is exactly comparable to the process we see in the furnace and the lamp. I will not here enter into an explanation of what is called the "initial impulse" which starts the combustion, the respiratory process whereby the oxygen is admitted and the dioxide of carbon is exhaled; but being started, like a fire, it continues to burn, as long as fuel (food) is supplied. If any one doubt that consciousness and all mental states are evolved from the aliment, within and by, the human machine, let him withhold all food and see how long consciousness will remain. It would be as reasonable, it seems to me, to speak of the immortality of digestion as of the immortality of the spirit, for the "spirit" is the breath, the function of respiration, the intake of oxygen, the output of carbon dioxide.

The question of soul—spirit, life,—of consciousness, is not transcendental. It is purely a physiological problem,—partly already solved in the biological laboratory. When solved it will be found to fall within the domain of physics and chemistry, and come under the laws which govern molecular mechanics. There are no "two minds." I am not acquainted with any authority that admits the postulate upon which Mr. Hudson bases his elaborate "Laws of Psychic Phenomena," unless I except Maudsley, and I think he was laboring under a delusion, just as, in my opinion, Mr. Hudson is.

There are many phases of one mind, consciousness being fundamental. Consciousness is a part of the higher activities of the mind (soul?), and, as such, is dependent on the normal structure of its psychic organ, the brain.

The most important discovery in the anatomy and physiology of the brain in recent years is that of the organs of thought. (Flechsig.) "They are the real organs of mental life; the highest instruments of psychic activity that produce thought and consciousness. But, given the thought organ, *how* is thought produced? At death their psychic activity is extinguished like every other physiological function." (Haeckel.) This, and other discoveries and advances along the lines of biological (zoological) research, and in general physics have, in the scientific mind; destroyed the myth of "spiritism"; but blind faith, faith in most preposterous things, faith in "miracles"—by heredity and ceaseless iteration and reiteration by tongue and pen, is so firmly fixed in the mind of the great majority, the enlightened (on all else) as well as the unenlightened, that reason will never uproot it.

Mrs. Piper's exhibitions of a wonderful faculty as little understood by herself as by any one else, defy, in the present state of knowledge, a satisfactory explanation. Clairvoyance and clairaudience, Dr. R. M. Bucke, of London, Ontario (Evolution of the Human Mind, *New York Medical Record*), calls "the dawning of a sixth sense." I, of course, offer not a suggestion of an explanation. I incline to the belief, however, that "telepathy" gives a cue which, like the mythical thread given Theseus by the beautiful Ariadne, would, when he shall have slain the Minotaur, lead him out of the Cretan labyrinth, may guide us to light. I am satisfied in my own mind that, however inexplicable all the manifestations of this mysterious faculty, all the so-called psychic phenomena witnessed at her seances are,—they were the emanations from a living brain, present or absent.

Regarding mind as an electrical form of energy, a force which expresses itself in such puzzling phenomena, a mode of molecular motion, waves—vibrations—bearing a strong resemblance to the electrical energy generated artificially, and which the immortal Marconi has demonstrated can be projected through space and awaken response on the part of a suitable receiver 2000 miles away, I see no reason why, at no remote day, the laws that govern what we all are bound to recognize as thought transference, clairvoyance, etc., should not be revealed to man by investigation along this line. The gateway has been opened by Helmholz and Hertz; the possibility of transmission of electrical energy through space has been demonstrated by the great Marconi, "physic phenomena" may, in time, be explained upon the principle and in accordance with the laws that govern all electrical phenomena.

I append two quotations quite apropos of the subject, and with which may views are entirely in accord.

"The principle of energy, and the correlative idea of the unity of natural forces on the basis of a common origin are now accepted by all competent physicists, and are regarded as the greatest advance of physics in the nineteenth century."—Haeckel, *Riddle of the Universe.*

"The phenomena of life can and should be explained solely by the working of the physical forces which control the material universe. Among those forces electricity plays a predominating part." (Ernest Solvay, Address at Brussels, December, 1893.)

[In an article like this, which must conform to the requirements of the occasion (a symposium, etc., "views as to psychic phenomena, tersely expressed"), I can not go into a discussion of the question raised by other contributors as to the nature and limitations of telepathy; whether it is limited to two persons, or may be transmitted through several living brains. Nor can I even allude to the details of electrical energy as generated in the living animal organism—and its mode of transmission; nor touch Faraday's (now accepted) theory of electrified or vitalized atoms.]

